# Preparation and Properties of a Lightweight, High-Strength, and Heat-Resistant Rigid Cross-Linked PVC Foam

**DOI:** 10.3390/polym15112471

**Published:** 2023-05-26

**Authors:** Kun Jiang, Yingchun Li, Heyun Wang, Hao Jia, Haoji Jiang, Hao Li, Ao Sheng

**Affiliations:** School of Chemistry and Chemical Engineering, Shihezi University, Shihezi 832003, Chinaliyingchun@163.com (Y.L.);

**Keywords:** rigid cross-linked PVC foam, 3-glycidoxypropyltriethoxysilane, high strength, semi-interpenetrating network, glass transition temperature, heat resistance

## Abstract

A rigid poly(vinyl chloride) foam with a cross-linked network structure was prepared by adding 3-glycidoxypropyltriethoxysilane (KH-561) into the universal formulation. The resulting foam had excellent heat resistance because of the increasing degree of cross-linking and number of Si–O bonds with a high heat resistance. The as-prepared foam was verified using Fourier-transform infrared spectroscopy (FTIR), energy-dispersive spectrometry (EDS) and foam residue (gel) analysis, which demonstrated that KH-561 was successfully grafted and cross-linked on the PVC chains. Finally, the effects of different KH-561 and NaHSO_3_ additions on the mechanical properties and heat resistance of the foams were studied. The results showed that the mechanical properties of the rigid cross-linked PVC foam were raised after adding a certain amount of KH-561 and NaHSO_3_. The residue (gel), decomposition temperature, and chemical stability of the foam significantly improved compared to the universal rigid cross-linked PVC foam (T_g_ = 72.2 °C). The T_g_ of the foam could reach 78.1 °C without any mechanical degradation. The results have important engineering application value regarding the preparation of lightweight, high-strength, heat-resistant, and rigid cross-linked PVC foam materials.

## 1. Introduction

Low-density rigid cross-linked PVC foams with unique gas–solid two-phase and cross-linked network structures have many excellent properties [1]. They are lightweight and have a high strength. They insulate against sound and heat with no water absorption. They have good chemical and fire resistance at a low cost. Such foams have been widely used in various fields as sandwich composites, including in wind energy, aerospace, ships, rail transit, sustainable buildings, etc. [2,3,4,5,6,7,8,9]. The heat distortion temperature (glass transition temperature) of a universal rigid cross-linked PVC foam material is 70 °C, which means that such a foam material can only be used in an environment below 70 °C. However, wall insulation, aerospace, and other applications often require higher temperatures [10,11]. Therefore, the preparation of rigid cross-linked PVC foam materials with a high glass transition temperature has important engineering value.

There are three approaches to preparing rigid cross-linked PVC foam materials. One method was mentioned by Yvan Landler (Kleber Colombes company), who proposed using the unsaturated acid anhydride and vinyl monomer co-polymerization under the action of the initiator conditions, grafting onto PVC macro-molecule chains, and then cross-linking the acid anhydride and isocyanate under the action of water, thus forming a cross-linked network [12]. However, the introduction of a vinyl monomer (styrene or acrylonitrile) can produce serious toxicity and combustion problems. The second method proposed for the preparation of a rigid cross-linked PVC foam involves the use of isocyanate and anhydride under the action of water to form a cross-linked network. The cross-linked net of isocyanate and anhydride constitutes a semi-interpenetrating network structure with a PVC matrix when the foam is forming [13]. The third method uses isocyanate and epoxy resin (E51) in the absence of anhydride composition to form a semi-interpenetrating network structure with a matrix resin PVC [14,15].

To improve the heat resistance of rigid cross-linked PVC foam, some researchers have introduced asphalt into the rigid cross-linked PVC foam, and the results have shown that the thermal deformation temperature (glass transition temperature) of the rigid cross-linked PVC foam can be increased to 75 °C. However, the asphalt material is soft, and it is difficult to mix the raw materials into a paste material. Shi [16] introduced trimethylolpropane trimethacrylate and triallyl isocyanurate in order to cross-link polymer chains on poly(vinyl chloride) molecules, thus improving the cross-linking degree of foam material, and obtaining a heat-resistant foam material with good mechanical properties.

Phthalic anhydride (PA) is usually used in commercial rigid cross-linked PVC foams. The polyamide network formed via the reaction of phthalic anhydride with excessive isocyanate in the form of an amide structure exists in the cross-linked network. Jiang [17] used the reaction of phthalic anhydride and isocyanate to confirm that the presence of phthalic anhydride leads to the formation of imide defect structures in rigid cross-linked PVC foam, which reduce the gel content and decrease the mechanical properties and heat resistance of the material.

The main methods used to improve the heat resistance of PVC involve the modification of the cross-linking of the materials [18]. Common modifiers are organosilanes [19,20,21], peroxides [20,22], triazine compounds [23], isocyanates [24], etc. Studies on organosilanes, peroxides, and triazines have mostly focused on the cross-linking of flexible PVC foams, while cross-linking studies on rigid PVC foams are relatively rare. It is well known that epoxy-based compounds are typical non-metallic stabilizers for PVC because the epoxy group can react with unstable chlorine on the PVC chain to form an ether bond, thereby preventing the formation of long-chain olefins, and that the compounds are the acceptor of hydrogen chloride [25,26]. As one of the epoxy compounds, 3-glycidoxypropyltriethoxy silane (KH-561) not only has grafting stability, but also can hydrolyze and condensate to form a cross-linked structure because of the presence of siloxane groups in the structure. Therefore, epoxy silane KH-561 has been introduced to form a cross-linked network structure of rigid PVC foam on the basis of the general formula (PA). Through the grafting and cross-linking of KH-561 between PVC molecular chains, excess cyanate ester was reacted with the active H in the cross-linking network to connect the anhydride and isocyanate in a cross-linking network, with KH-561 grafted and cross-linked onto the PVC network. The introduction of KH-561 can enhance the cross-linking of materials and introduce heat-resistant Si–O bonds into the chains to improve the heat resistance of the foam materials. There are many studies on the heat resistance of PVC that involve the grafting of silane to form cross-linked structures. The cross-linking mechanism is well known, but it has not been used in the preparation of low-density rigid cross-linked PVC foams.

In this study, epoxysilane was introduced into the universal rigid cross-linked PVC foam formulation, and a new method for preparing heat-resistant rigid cross-linked PVC foam in the presence of epoxysilane was explored. The chemical structure of the rigid cross-linked PVC foam that was modified by epoxysilane was studied in detail. The possible chemical reactions in the preparation process and the effect of the addition of epoxy silane on the heat resistance and mechanical properties of the foam material were further studied.

## 2. Materials and Methods

### 2.1. Materials

Poly(vinyl chloride) (PVC) paste resin was purchased from Xinjiang Tianye (Group) Co., Ltd., Xinjiang, China. Toluene diisocyanate (TDI) was purchased from Bayer AG, Leverkusen, Germany. Phthalic anhydride (PA) was purchased from Shanghai Macklin Biochemical Co., Ltd., Shanghai, China. Azodicarbonamide (AC) was purchased from ShanghaiRyon Biological Technology Co., Ltd., Shanghai, China. 2,2-azodiisobutyronitrile (AIBN) was purchased from Chengdu Kelon Chemical Reagent Factory, Chengdu, China. Liquid barium–zinc composite stabilizer was purchased from Shenzhen VINOVA Chemicals Co., Ltd., Shenzhen, China. The 3-glycidoxypropyltriethoxysilane (KH-561) was purchased from Jining Huakai Resin Co., Ltd., Jining, China. Sodium hydrogen sulfite (NaHSO_3_) was purchased from Tianjin Shengao Chemical Reagent Co., Ltd., Tianjin, China.

### 2.2. Preparation of Rigid Cross-Linking PVC Foams

**Figure 1:**
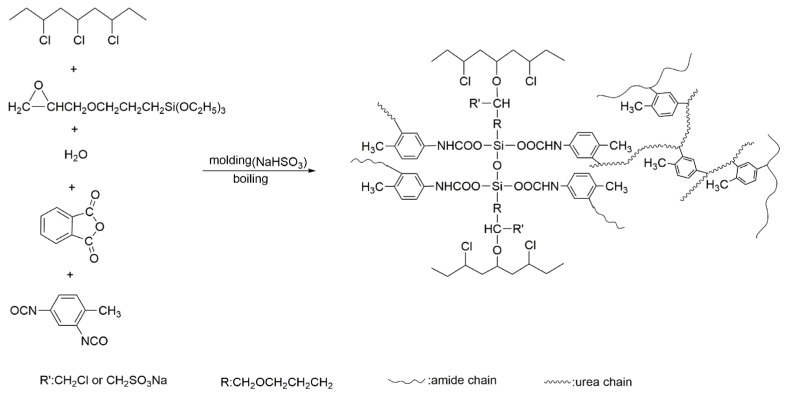
Schematic illustration of grafting and cross-linking processes of rigid cross-linked PVC foam materials.

Table 1 shows the formulation of the universal rigid cross-linked PVC foam material and the addition of KH-561 used to prepare a new kind of rigid cross-linked PVC foam material. The method used to prepare the rigid cross-linked PVC foam material is as follows: First, the raw materials were fully mixed and stirred to form a homogenous paste. Second, the paste material was poured into the mold (220 mm × 220 mm × 15 mm). The mold was pressed for 20 min at 170 °C and 15 MPa, and then cooled, opened and the pressure was relieved to obtain an elastic porous body. PVC resin gels were formed under the condition of heating and pressurizing, and all solid components were evenly dispersed in the continuous phase of the PVC resin gel. The solid foaming agent was decomposed, with heat rising to generate gas, and was dispersed to form initial porous materials. This offered bubble nuclei for subsequent foaming. Third, the resulting elastic porous body was boiled and foamed in a water bath at 92 °C for 3 h, and then placed in an oven at 80 °C for 2 h post-treatment to obtain a rigid cross-linked PVC foam material. Based on the different amounts of KH-561, NaHSO_3_ and PA added to the formulation, the samples were named as PKN0-0, PKN3-1, PKN6-2, PKN6-0.6, PKN6-0, PKN9-3, PKN12-4 and PKN3-1-0. Figure 1 shows the schematic diagram of the grafting and cross-linking of the rigid cross-linked PVC foam materials.

### 2.3. Characterization

The gel content can reflect the degree of cross-linking in the cross-linked structure in the PVC foam to a certain extent. Thus, 0.2 g of the foam material was weighed, and the sample was wrapped in a stainless mesh, placed in a cable extractor, and extracted using tetrahydrofuran (THF) for 36 h. After the end of the extraction period, the solid (F-F-S) was obtained by extracting THF, and the liquid substance (F-F-L) was obtained by evaporating the extract. These were dried to constant weight, and the gel content (GC) was calculated according to Equation (1).

(1) $$\mathrm{GC}=\frac{m_{1}}{m_{2}}\times 100\%$$

Here, m_1_ is a solid obtained via extraction from dry to constant weight in THF, and m_0_ is the mass of the foam.

The glass transition temperature of the rigid cross-linked PVC foam was analyzed using a DSC-200F3 differential scanning calorimeter under a nitrogen atmosphere at a heating rate of 10 K/min from 20 °C to 140 °C.

The morphology of the cells in the molded block and foam was studied using JSM-6490LV scanning electron microscopy (SEM).

The FTIR spectra were collected from 4000–500 cm^−1^ at a resolution of 4 cm^−1^ using a Nicolet 360 FTIR spectrometer. The different samples were mixed and ground with KBr powder, pressed into tablets, and analyzed.

The elemental analysis of the extracted solids of the foam material in the observed area was revealed using energy-dispersive spectrometry (EDS) attached to the SEM.

TGA was performed on a Netzsch STA 449F3 instrument. Samples (5–8 mg) were heated from 50 °C to 600 °C at 10 K/min under flowing nitrogen.

The mechanical properties of the rigid cross-linked PVC foam samples were tested using an Instron 3366 tester. The compression properties and tensile properties of the foam were tested according to ASTM D1621 and C297 standards, respectively [27,28,29,30]. The samples were cut to size as specified in the standard, and then the compression properties and tensile properties were tested by Instron 3366 with a loading rate of 2.5 mm/min. All property data were tested five times and were analyzed statistically using the Student’s *t* test.

## 3. Results and Discussion

### 3.1. SEM Analysis of Rigid Cross-Linked PVC Foams

**Figure 2:**
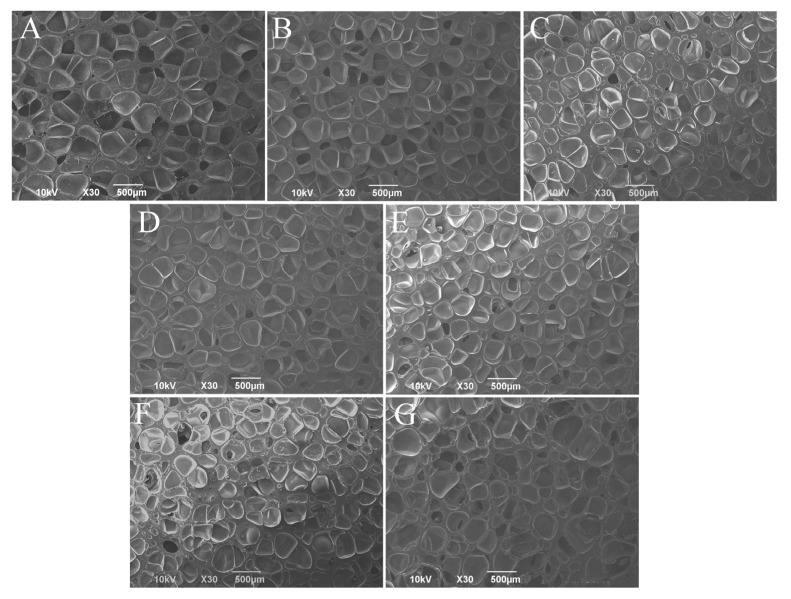
SEM micrographs (×30 magnification) of the molding blocks with different KH-561 and NaHSO_3_ contents. ((**A**): PKN0-0, (**B**): PKN3-1, (**C**): PKN6-2, (**D**): PKN6-0.6, (**E**): PKN6-0, (**F**): PKN9-3, (**G**): PKN12-4).

Figure 2 shows the SEM images of the mold blocks pressed for 20 min under the conditions of 170 °C and 15 MPa for uniform pastes with different amounts of KH-561 and NaHSO_3_. The mold blocks PKN0-0, PKN3-1, PKN6-2, PKN6-0.6, and PKN6-0 are filled with spherical bubbles of uniform size, and the bubbles are evenly distributed in the mold block, indicating that the foaming agents AC and AIBN undergo thermal decomposition and generate gas under a high temperature and pressure. However, the bubble size of the molded block PKN12-4 is uneven because a large number of small molecules of KH-561 and NaHSO_3_ can reduce the melting strength of the molding block so that the gas in the melt easily forms bubble nuclei and increases the size of the bubble nucleus.

**Figure 3:**
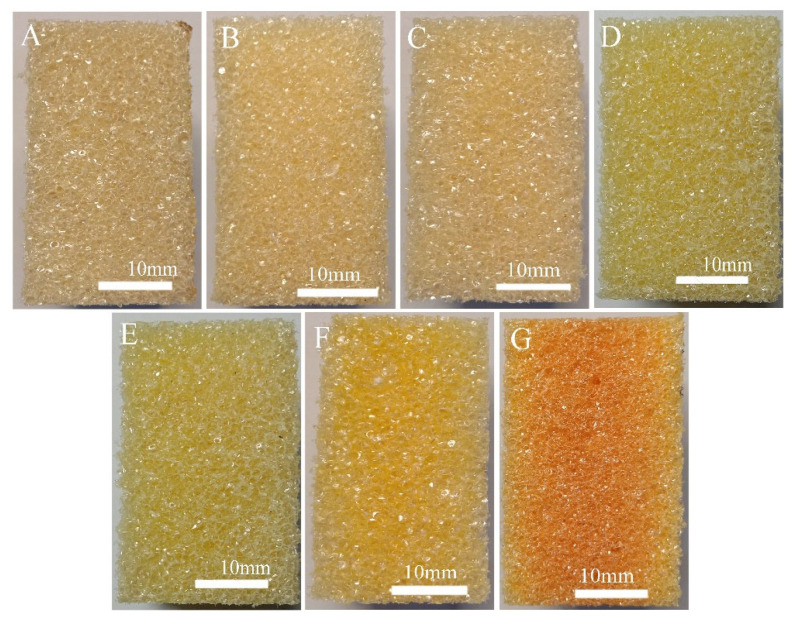
Photographs of rigid cross-linked PVC foam materials with different KH-561 and NaHSO_3_ contents. ((**A**): PKN0-0, (**B**): PKN3-1, (**C**): PKN6-2, (**D**): PKN6-0.6, (**E**): PKN6-0, (**F**): PKN9-3, (**G**): PKN12-4).

**Figure 4:**
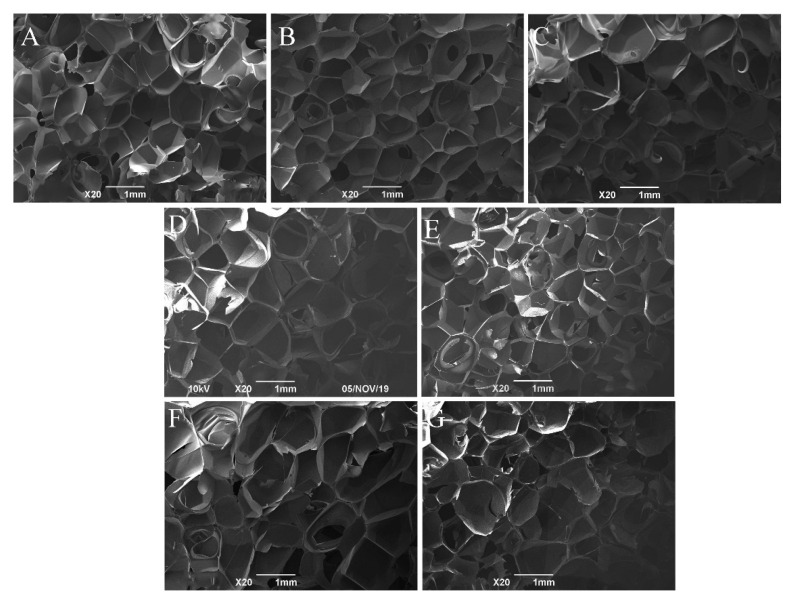
SEM micrographs (×20 magnification) of rigid cross-linked PVC foam materials with different KH-561 and NaHSO_3_ contents. ((**A**): PKN0-0, (**B**): PKN3-1, (**C**): PKN6-2, (**D**): PKN6-0.6, (**E**): PKN6-0, (**F**): PKN9-3, (**G**): PKN12-4).

The properties of foam materials depend not only on the chemical structure and aggregate structure of polymer matrix materials, but also on the cell morphology of the foam [31]. Figure 3 and Figure 4 show photos and SEM images of the rigid cross-linked PVC foam materials with different KH-561 and NaHSO_3_ contents. Figure 3 and Figure 4 show that the foam materials PKN3-1, PKN6-2, PKN9-3, and universal foam PKN0-0, added with a certain proportion of KH-561 and NaHSO_3_, have a similar bubble size, bubble shape, and thin bubble wall characteristics. The density of the resulting foam materials is (40 ± 4) kg/m^3^. The bubble shape and size of foam materials PKN6-2, PKN6-0.6, and PKN6-0 did not change significantly as the amount of NaHSO_3_ decreased; the density of the foam materials was also similar. However, the foam material PKN12-4 is brick red, and the cell appears to collapse and coalescence. This is because the amount of KH-561 added is too large, and a large amount of KH-561 (which has not undergone a grafting reaction) reduces the attraction between the PVC molecules. This makes the resin soft, reduces the processing temperature of the resin, reduces the melting strength of the molded block, and reduces the growth resistance of the bubble body. However, the results also show that the density of the rigid cross-linked PVC foam with different KH-561 and NaHSO_3_ contents is not significantly different. Therefore, the mechanical properties of the foamed plastics are mainly affected by the matrix cross-linked structure.

### 3.2. FTIR Analysis of Rigid Cross-Linked PVC Foams

**Figure 5:**
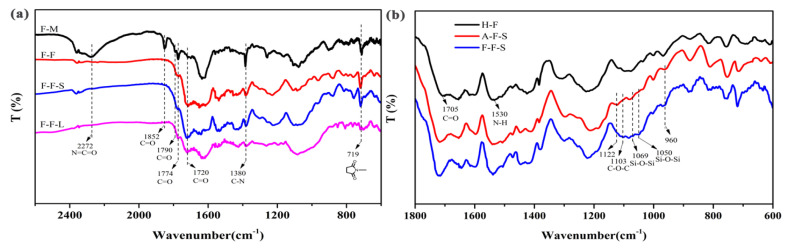
(**a**) Fourier transform infrared (FTIR) spectra of F foam: molded block (F-M), F foam (F-F), extraction of solids (F-F-S) and liquids (F-F-L) from F foam; (**b**) Zoom-in FTIR spectra of H foam, extraction solids of A foam (A-F-S) and extraction solids of F foam (F-F-S) from 600–1800 cm^−1^.

In a previous study on the preparation of rigid cross-linked PVC foam materials, the molding process, as the gelation of the PVC resin and the thermal decomposition of the foaming agent to produce gas, was shown. Chemical reactions other than the thermal decomposition of the blowing agent are rarely discussed. Infrared research on the various stages of the foam preparation process was conducted. Figure 5 shows the infrared spectrum of the different stages in the preparation of foam materials.

The FTIR spectra of the F-M show the C=O symmetrical and asymmetric absorption peaks of PA at 1852 cm^−1^, 1790 cm^−1^, and 1774 cm^−1^, and the N=C=O characteristic absorption peaks of isocyanate at 2272 cm^−1^. However, the characteristic absorption peaks of the phthalimide structure appear at 719 cm^−1^, and these correspond to the characteristic absorption peaks of a five-member cyclic imine. This fully shows that isocyanate reacts with phthalic anhydride to produce imide [32] in the process of molding. The KH-561 cross-linked modified foam F-F shows a C=O stretching vibration peak of acyclic monoalkyl urea at 1601 cm^−1^, as well as the C=O stretching vibration peak and N–H bending vibration of amide at 1655 cm^−1^ and 1541 cm^−1^, respectively. The C=O characteristic absorption peaks of PA are at 1852 cm^−1^, 1790 cm^−1^, and 1774 cm^−1^, and the N=C=O characteristic absorption peak of isocyanate at 2272 cm^−1^ disappeared. The FTIR of F-F shows that the anhydride and isocyanate completely participate in the formation of the cross-linked network under the action of water during boiling. Compared to the extraction solids of the universal foam materials in Figure 5b, the extraction solids of the KH-561-modified cross-linked PVC foam materials F show a wide peak between 960 cm^−1^ and 1121 cm^−1^. This is because the absorption peak of the bond between Si–O–Si (1050 cm^−1^ and 1069 cm^−1^) [19,33], and C–O–C (1100 cm^−1^) appears, which has a wider peak. This suggests that the silane was successfully grafted and cross-linked onto the PVC chains. During the boiling process, a large amount of Si–OH was formed by the hydrolysis of silane, and excessive NCO reacted with Si–OH to form carbamate. According to Figure 5b H-F, the characteristic absorption peaks of the carbamate group C=O bond and N–H bond appear at 1705 cm^−1^ and 1530 cm^−1^, which explains the formation of carbamate. However, the infrared spectrum of F-F-L shows the characteristic absorption peaks of imide at 1774 cm^−1^, 1720 cm^−1^, 1380 cm^−1^, and 719 cm^−1^, which fully show that part of the cross-linked structure can be extracted via THF. The results showed that the KH-561-modified cross-linked foam has the structure of imides, amides, urea, carbamate, and Si–O–Si after boiling.

### 3.3. EDS Analysis of Rigid Cross-Linked PVC Foams

Table 2 uses the energy-dispersive spectrometer attached to the scanning electron microscope to analyze the composition of the solids extracted from foams. Table 2 shows that the Si and Cl in the solids extracted from foam materials PKN3-1, PKN6-2, and PKN9-3 increase as the silane content increases. This indicates that NaHSO_3_ can improve the grafting reaction between KH-561 and PVC chains and can improve the grafting and cross-linking degree of the material. The content of Si in the solids extracted from foam materials PKN6-2, PKN6-0.6, and PKN6-0 stabilized as the NaHSO_3_ content decreased, and the content of Cl decreased as the NaHSO_3_ content decreased, which indicated that the silane had successfully reacted with the cross-linking network. Infrared analysis shows that the addition of a fixed proportion of KH-561 and NaHSO_3_ can successfully graft and cross-link onto PVC macro-molecular chains. The PVC molecular chains exist in a cross-linking network and can improve the cross-linking degree of the PVC foam.

**Scheme 1:**
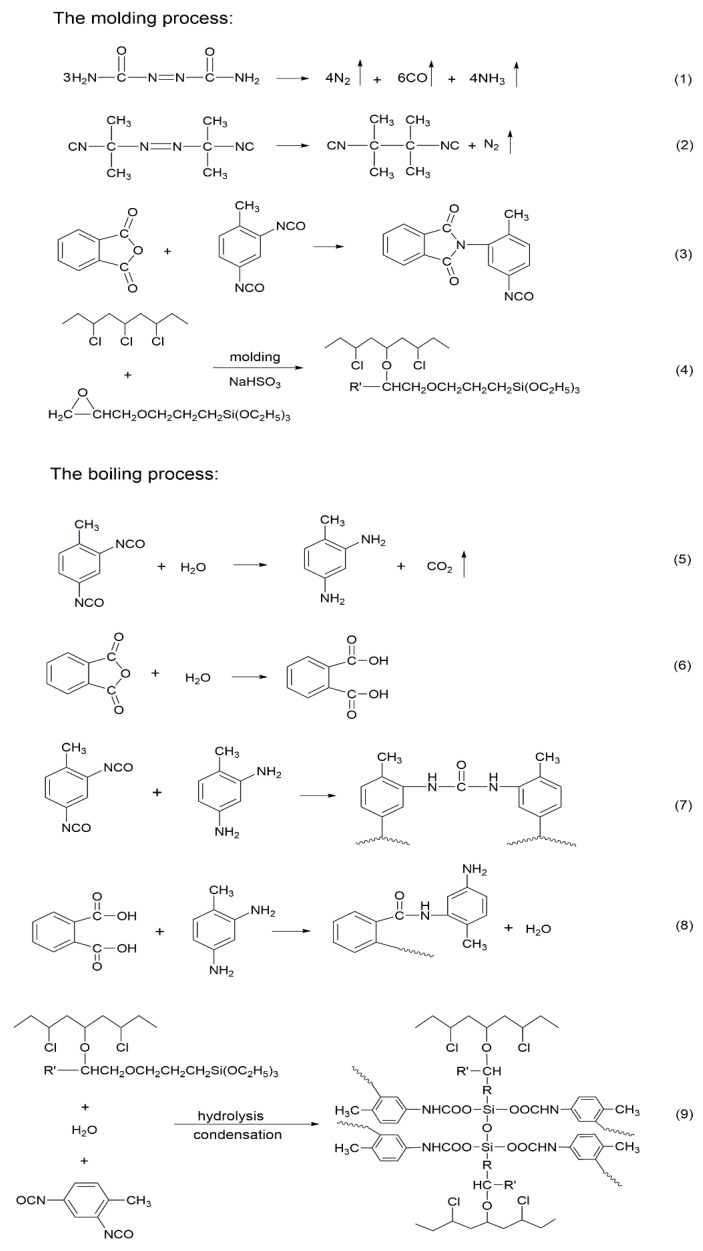
Possible reactions during the preparation of rigid cross-linked PVC foam materials.

The preparation of rigid cross-linked PVC foams involves a reaction between KH-561, PVC, isocyanate, anhydride, and water. Due to the FTIR and EDS analysis of the KH-561 cross-linked and modified PVC foam sample with regard to the rigid cross-linked PVC foam materials comprising a semi interpenetrating network structure, a KH-561 grafted and cross-linked PVC chain was proposed, and it was found that excessive isocyanate reacted with hydrolyzed KH-561. The detailed reaction process is shown in Scheme 1. First of all, during the molding process, the blowing agent is thermally decomposed to generate a gas to form a bubble core. At the same time, KH-561 grafts onto the PVC molecular chains under the action of NaHSO_3_, and the isocyanate reacts with the acid anhydride to form an imide. During boiling, KH-561 is hydrolyzed and condensed to form a cross-linked structure. The isocyanate is then reacted with water to produce corresponding amines, while releasing CO_2_. Anhydride is hydrolyzed to produce corresponding acids. Excess isocyanate is reacted with acids and amines with high activity, forming amides and urea, respectively. Excessive NCO reacts with the Si–OH produced by the hydrolysis of KH-561 to form carbamate.

### 3.4. Gel Content Measurement

The gel content can reflect the cross-linking degree of the cross-linking network. It is well known that the universal rigid cross-linked PVC foam is based on isocyanate and phthalic anhydride as a network backbone. During the boiling process, excessive isocyanate cross-links the network to form a cross-linked network in which PVC linear molecular chains are interspersed in the polymer network. The PVC linear molecular chain can be extracted using THF. In this study, the gel content of the rigid cross-linked PVC foam modified by KH-561 was tested to study the influence of the addition of KH-561 on the content of foam gel.

As shown in Table 3, the gel content of the rigid cross-linked PVC foams PKN3-1, PKN6-2, PKN9-3 and PKN3-1-0 was 41.92 ± 0.58%, 44.25 ± 0.71%, 46.22 ± 0.76%, 47.55 ± 0.26% after adding 3phr, 6phr, 9phr and 12phr KH-561 based on the universal rigid cross-linked PVC foam. Compared with the gel content of universal foam PKN0-0 (37.20 ± 0.10%), the gel content of the foam materials showed an upward trend with an increase in the fixed ratio of KH-561 and NaHSO_3_. The foam materials PKN6-2, PKN6-0.6, and PKN6-0 show that the gel content of the foam decreases as the amount of added NaHSO_3_ decreases. The gel contents of PKN9-3 and PKN12-4 increased rapidly by 24.24% and 27.82% compared toPKN0-0, with an increase in the ratio of KH-561 and NaHSO_3_. This indicates that the addition of NaHSO_3_ can promote the silane to graft PVC and improve the cross-linking degree. Because the KH-561 molecules are grafted onto the PVC chains via a nucleophilic substitution reaction, increasing the electronegativity of KH-561 improves its grafting reaction with PVC; meanwhile, NaHSO_3_ is a strong-alkali weak-acid salt, which can interact with KH-561 to increase the oxygen’s electronegativity.

### 3.5. Static Thermal Stability Analysis of Rigid Cross-Linked PVC Foams

**Figure 6:**
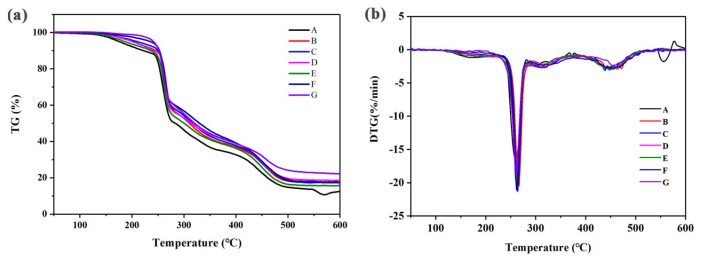
TG curves (**a**) DTG curves (**b**) of rigid cross-linked PVC foam with different KH−561 and NaHSO_3_ contents. (A: PKN0−0, B: PKN3−1, C: PKN6−2, D: PKN6−0.6, E: PKN6−0, F: PKN9−3, G: PKN12−4).

Figure 6 shows the TG and DTG analysis of the thermal degradation of rigid cross-linked PVC foam materials with different amounts of KH-561 and NaHSO_3_ under N_2_. The TG–DTG analysis of the rigid cross-linked PVC foam materials with different amounts of KH-561 and NaHSO_3_ revealed three stages of decomposition. The first mass loss could be attributed to the evolution of the water and solvent retained in the rigid cross-linked foam materials. The second stage of decomposition was related to the degradation of PVC and carbamate. The parameters for the 5% weight loss temperature (T_5%_), 10% weight loss temperature (T_10%_), 50% weight loss temperature (T_50%_), and the residual rate at 600 °C are shown in Table 4. The thermal decomposition temperature (T_5%_) of universal PVC foams is 176 °C. The 5% weight loss temperature of foams PKN3-1, PKN6-2, PKN9-3, and PKN12-4 foams is significantly increased by adding a fixed ratio of KH-561 and NaHSO_3_ on the basis of the universal formula. The heat loss temperatures of 5% are 201 °C, 210 °C, 236 °C, and 242 °C, respectively. The thermal degradation data of foams PKN6-2, PKN6-0.6, and PKN6-0 show that the thermal decomposition temperature of the foams decreases as the NaHSO_3_ content decreases. The data analysis of the thermal degradation of rigid cross-linked PVC foams shows that adding KH-561 can absorb the hydrogen chloride released by PVC during heating, inhibit the further degradation of PVC, and improve the thermal stability of foams slightly. In the third decomposition stage, polyene sequences formed. The PKN3-1, PKN6-2, PKN6-0.6, PKN6-0, PKN9-3, PKN12-4 residuals were 17.2%, 17.1%, 18.5% 15.7%,17.5% and 22.1%, respectively, which were 10.8% higher than the pure PKN0-0 residual at 600 °C. These results demonstrated that the semi-interpenetrating network could promote charring during the degradation process. On the basis of silane addition, adding NaHSO_3_ can enhance the cross-linking degree of foam materials and increase the force between molecular chains. This makes the decomposition of small molecular chains more difficult, and can significantly improve the thermal stability of the foams. In addition, the residue of the KH-561-modified and cross-linked PVC foam is higher than the universal rigid cross-linked PVC foam, which indicates that the addition of KH-561 can prevent carbonization during degradation.

### 3.6. Glass Transition Temperature Analysis of Rigid Cross-Linked PVC Foams

The glass transition temperature (T_g_) is a relaxation phenomenon that occurs in amorphous polymers from the freezing state to the thawing state. It is an important characteristic parameter of polymer materials. In industry, the use temperature of polymer materials is determined by the T_g_ of the polymer.

**Figure 7:**
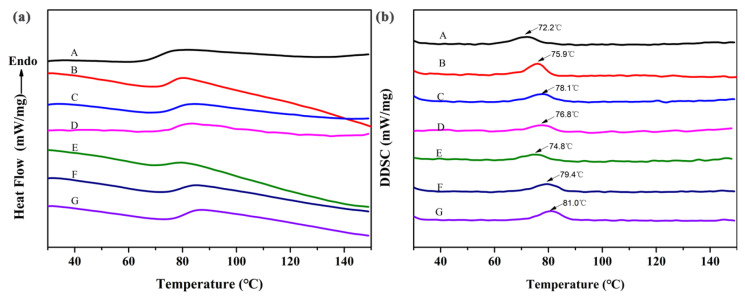
(**a**) DSC and (**b**) DDSC curves of rigid cross-linked PVC foam with different KH-561 and NaHSO_3_ contents. (A: PKN0-0, B: PKN3-1, C: PKN6-2, D: PKN6-0.6, E: PKN6-0, F: PKN9-3, G: PKN12-4).

Figure 7 shows the DSC and DDSC curves of the rigid cross-linked PVC foams with different KH-561 and NaHSO_3_ contents. The T_g_ of the rigid cross-linked PVC foams PKN3-1, PKN6-2, PKN9-3, and PKN12-4 increases with the addition of a fixed ratio of KH-561 and NaHSO_3_. The T_g_ of rigid cross-linked PVC foam PKN6-2 is about 6 °C higher than that of universal rigid cross-linked PVC foams, and guarantees the mechanical properties of the foams. The T_g_ of the foam increases to 81 °C when the amount of KH-561 is increased to 12 phr. The glass transition temperatures of foam materials PKN6-2, PKN6-0.6, and PKN6-0 decrease as the NaHSO_3_ content is decreased (78.1 °C, 76.8 °C, and 74.8 °C, respectively). This decrease is caused by the addition of NaHSO_3_, which facilitates the successful grafting of PVC macro-molecular chains onto the cross-linked network, and the resulting network structure hampers movement between the molecular chains.

### 3.7. Mechanical Properties of Rigid Cross-Linked PVC Foams

The mechanical properties of a foam are an important index for bearing materials in a sandwich structure. The mechanical properties of the foam depend not only on the foam’s cellular structure, but also on the chemical structure of the polymer matrix [34]. Table 5 shows the mechanical properties of the rigid cross-linked PVC foam that was prepared by adding different amounts of KH-561. The cells of the foam exhibit a similar morphology (Figure 5), and the differences in the mechanical properties of the foam cannot be attributed to the foam cell structure, but should be related to the chemical structure of the PVC foam matrix. Here, the density of the foam is slightly different, so the evaluation of the mechanical properties of the foam is based on the specific strength of the foam material. When the ratio of KH-561 to NaHSO_3_ is constant, the strength of foams PKN3-1, PKN6-2, KPN9-3, and KPN3-1-0 firstly increases and then decreases rapidly under unit density [35]. When the content of KH-561 reaches 9 phr, the compressive strength decreases to (0.2 ± 0.03) MPa. This is due to the introduction of too many flexible KH-561 segments in the cross-linking network, which can improve the movement ability of the PVC chains and reduce the rigidity of the PVC molecular chains; this reduces the compressive strength of the foam material. For the PKN6-2, PKN6-0.6, and PKN6-0 foam materials, the compressive strength and tensile strength were found to increase slightly relative to the unit density. Because the content of NaHSO_3_ decreases the ability to graft onto PVC molecular chains, the force on the matrix resin PVC molecules also decreases, and because KH-561 mainly exists in the polyurea–polyamide cross-linked network, the cross-linking entanglement of KH-561 makes it difficult to move the molecular chains of the cross-linked network. The specific tensile strengths of the foam materials PKN3-1, PKN6-2, and PKN9-3, made with a fixed proportion of KH-561 and NaHSO_3_, are 1.6 × 10^−2^, 1.6 × 10^−2^, and 1.5 × 10^−2^ MPa/(kg/m^3^)). These are slightly higher than the universal foam material PKN0-0. The comprehensive mechanical properties showed an increase in the tensile strength of foam materials with different amounts of added KH-561 and NaHSO_3_. The slight increase in the tensile strength is mainly attributable to the increase in the cross-linking degree of foams, the increase in the entanglement between molecular chains, and the increase in the chain-breaking resistance. These features make it easy to produce stress concentration when subjected to external forces.

## 4. Conclusions

The introduction of KH-561 improves the cross-linking degree of the PVC foam material and the heat resistance of the PVC foam materials. The results also verified that NaHSO_3_ could promote the KH-561 grafting and cross-linking of PVC molecular chains. The heat resistance of the PVC foam materials is related to the cross-linking degree of the material and the introduction of heat-resistant Si–O bonds in the network. Within a certain density range, the tensile properties of the foam materials changed little, and the compressive properties first increased and then decreased, with the increase in NaHSO_3_ and KH-561 added in a fixed proportion. The cross-linking of the PVC polymer chains makes the decomposition temperature and thermal stability of the foam materials higher than that of the universal rigid cross-linked PVC foam material, and the T_g_ of the rigid cross-linked PVC foam, when modified by KH-561 with the addition of NaHSO_3_ as an accelerator, can reach 81.0 °C. Therefore, the selection of a certain amount of KH-561 and NaHSO_3_ can improve the mechanical properties and heat resistance of the foam.

## Figures and Tables

**Table 1:** Formulas of rigid cross-linked PVC foam plastics.

Formulation	PKN0-0	PKN3-1	PKN6-2	PKN6-0.6	PKN6-0	PKN9-3	PKN12-4	PKN3-1-0
PVC	100	100	100	100	100	100	100	100
TDI	60	60	60	60	60	60	60	60
PA	25	25	25	25	25	25	25	0
AC	2	2	2	2	2	2	2	2
AIBN	3.7	3.7	3.7	3.7	3.7	3.7	3.7	3.7
Liquid barium zinc composite stabilizer	4	4	4	4	4	4	4	4
KH-561	—	3	6	6	6	9	12	3
NaHSO_3_	—	1	2	0.6	0	3	4	1

**Table 2:** EDS analysis results of extracted solids of foams made with differed KH-561 and NaHSO_3_ contents.

Sample	Element Atomic (%)					
	C	N	O	Cl	Si	Na
**PKN0-0**	66.24	14.38	12.50	6.81	0.03	0.04
**PKN3-1**	66.55	13.69	13.28	6.06	0.18	0.24
**PKN6-2**	62.46	12.63	12.96	11.17	0.31	0.47
**PKN6-0.6**	65.43	13.12	14.19	6.86	0.32	0.08
**PKN6-0**	65.36	12.80	14.11	7.38	0.31	0.03
**PKN9-3**	64.75	10.78	13.90	9.92	0.45	0.19

**Table 3:** Density and gel content of the rigid cross-linked PVC foam with different KH-561 and NaHSO_3_ contents.

Sample	Density (kg/m^3^)	Gel Content (%)
**PKN0-0**	43.3 ± 1.9	37.20 ± 0.10
**PKN3-1**	43 ± 2.0	41.92 ± 0.58
**PKN6-2**	36.9 ± 2.4	44.25 ± 0.71
**PKN6-0.6**	42.7 ± 2.0	38.63 ± 0.12
**PKN6-0**	42.8 ± 2.3	37.80 ± 0.22
**PKN9-3**	40 ± 1.7	46.22 ± 0.76
**PKN12-4**	42 ± 2.0	47.55 ± 0.26

**Table 4:** Thermal decomposition results for the different KH-561 and NaHSO_3_ content foams.

Sample	T_loss5%_ (°C)	T_loss10%_ (°C)	T_los50%_ (°C)	Residue (%)
**PKN0-0**	176	221	285	10.8
**PKN3-1**	201	246	311	17.2
**PKN6-2**	210	251	316	17.1
**PKN6-0.6**	200	240	315	18.5
**PKN6-0**	187	236	300	15.7
**PKN9-3**	236	253	330	17.5
PKN12-4	242	252	320	22.1

**Table 5:** Mechanical properties of foam materials made with different KH-561 and NaHSO_3_ contents.

Sample	Compressive Strength (MPa)	Specific Compressive Strength [MPa/(kg/m^3^)]	Tensile Strength (MPa)	Specific Tensile Strength [MPa/(kg/m^3^)]
**PKN0-0**	0.39 ± 0.03	9.0 × 10^−3^	0.56 ± 0.01	1.3 × 10^−2^
**PKN3-1**	0.42 ± 0.02	9.8 × 10^−3^	0.69 ± 0.05	1.6 × 10^−2^
**PKN6-2**	0.34 ± 0.02	9.2 × 10^−3^	0.59 ± 0.01	1.6 × 10^−2^
**PKN6-0.6**	0.43 ± 0.01	1.0 × 10^−2^	0.62 ± 0.01	1.5 × 10^−2^
**PKN6-0**	0.46 ± 0.07	1.1 × 10^−2^	0.91 ± 0.01	2.1 × 10^−2^
**PKN9-3**	0.2 ± 0.03	5.0 × 10^−3^	0.59 ± 0.02	1.5 × 10^−2^
PKN3-1-0	0.2 ± 0.04	4.8 × 10^−3^	----	----

## Data Availability

The authors declare that the data contained in this article is true and reliable.
